# Growth suppression by MYC inhibition in small cell lung cancer cells with TP53 and RB1 inactivation

**DOI:** 10.18632/oncotarget.8826

**Published:** 2016-04-18

**Authors:** Francesco Paolo Fiorentino, Elvan Tokgün, Sònia Solé-Sánchez, Sabrina Giampaolo, Onur Tokgün, Toni Jauset, Takashi Kohno, Manuel Perucho, Laura Soucek, Jun Yokota

**Affiliations:** ^1^ Genomics and Epigenomics of Cancer Prediction Program, Institute of Predictive and Personalized Medicine of Cancer (IMPPC), Campus Can Ruti, Barcelona, Spain; ^2^ Vall d'Hebron Institute of Oncology (VHIO) Hospital Vall d'Hebron, Barcelona, Spain; ^3^ Division of Genome Biology, National Cancer Center Research Institute, Tokyo, Japan; ^4^ Catalan Institution for Research and Advanced Studies (ICREA), Barcelona, Spain; ^5^ Department of Biochemistry and Molecular Biology, Universitat Autònoma de Barcelona, Bellaterra, Spain

**Keywords:** small cell lung cancer, MYC, CDKN1A, MYCL, SCLC

## Abstract

Small cell lung cancer (SCLC) is the most aggressive type of lung cancer with high mortality. One of the *MYC* family genes, *MYC*, *MYCL* or *MYCN,* is amplified in ~20% of the SCLCs; therefore, MYC proteins are potential therapeutic targets in SCLC patients. We investigated the therapeutic impact of Omomyc, a MYC dominant negative, in a panel of SCLC cell lines. Strikingly, Omomyc suppressed the growth of all tested cell lines by inducing cell cycle arrest and/or apoptosis. Induction of G1 arrest by Omomyc was found to be dependent on the activation of *CDKN1A*, in part, through the TP73 pathway. Our results strongly indicate that SCLC cells carrying amplification of *MYC*, *MYCL* or *MYCN* are addicted to MYC function, suggesting that MYC targeting would be an efficient therapeutic option for SCLC patients.

## INTRODUCTION

Small cell lung cancer (SCLC) is the most aggressive type of lung cancer with only 5% of five-year survival rate after diagnosis [1]. This is in part due to the fact that proper targeting therapies for SCLC have not yet been developed. In SCLCs, only a limited number of genes, such as *TP53* and *RB1*, are recurrently mutated [2–5]. One of the *MYC* family genes, *MYC*, *MYCL* or *MYCN*, is amplified and overexpressed in ~20% in a mutually exclusive manner and represents the most prominent activating oncogene alteration in SCLC [2, 4, 6]. Therefore, MYC proteins are strong candidates as therapeutic targets in patients with SCLC. However, the following crucial points must be taken into account. In mice, functional inactivation of *TP53* together with *RB1* is sufficient for the development of SCLC, and *MYCL* amplification occurs during SCLC progression [7, 8]. Similarly, in humans, *MYC* amplification is also likely to occur during SCLC progression [2, 4, 6]. While reconstitution of either *TP53* or *RB1* induces G1 arrest and apoptosis in human SCLC cell lines [9, 10], it is not clear whether MYC suppression is sufficient to inhibit SCLC cell growth. Consequently, if the growth of human SCLC cells is not dependent on amplified *MYC* family genes, MYC suppression would not be sufficient to have any therapeutic effect. In several mouse models of MYC-driven cancers, tumor regression by MYC suppression was hampered by the concomitant repression of TP53 or RB1 proteins, which highlighted the relevance of intact *TP53* and *RB1* pathways for the treatment of cancer by MYC targeting [11–13]. In addition, since MYC proteins are overexpressed in SCLC cells, higher dose of MYC inhibitor administration would be required than in cancer cells without *MYC* family genes amplification. Alternatively, it is also possible that MYC suppression could be highly effective if SCLC cells are addicted to the expression of amplified *MYC* family genes.

Mutually exclusive amplification of the three *MYC* family genes and the concurrent expression of two or three *MYC* family genes together, even though only one of them is amplified [14], imply the convenience of a common suppressing agent to all MYC proteins, MYC, MYCL and MYCN, to inhibit the growth of SCLC cells by MYC inhibition. MYC proteins are transcription factors with highly conserved and functionally important regions organized in a similar manner among the three paralogs [15]. DNA-binding activity depends on a ~100 amino-acid carboxy-terminal region comprising the basic helix-loop-helix leucine zipper (bHLH-LZ) domain that confers MYC proteins a highly specific interaction with another factor, MAX. The heterodimer MYC-MAX binds DNA at E-Box sequences to drive transcription of numerous target genes. Furthermore, the MYC-MAX dimeric bHLH-LZ region forms a platform for the binding of other factors, such as MIZ1 (ZBTB17), to repress transcription of a set of genes which share the initiatior (Inr) element at their promoter region [16]. Intriguingly, it has been recently reported that *MAX*-inactivating alterations occur in ~6% of SCLCs in a mutually exclusive manner to amplification of *MYC* family genes, highlighting the relevance of MYC pathway in SCLC progression [17]. Soucek et al. developed a dominant-negative MYC, termed Omomyc, containing MYC bHLH-LZ domain with four amino acid substitutions that confer high binding affinity to both MYC and MAX, as well as MYCN [18–20]. By competitive binding to both MYC and MAX, Omomyc prevents MYC-MAX heterodimerization and their interaction with the E-box. Consequently, overexpression of Omomyc inhibits the binding of MYC to DNA and transcription of *MYC* target genes [20, 21]. Omomyc induces apoptosis and/or mitotic defects in MYC-driven papillomatosis [21], lung adenocarcinoma [22, 23], SV40-driven insulinoma [24], and glioblastoma [25]. Therefore, Omomyc is an efficient inhibitor of both MYC and MYCN. Although inhibition of MYCL by Omomyc has not been investigated, based on the similarity of MYCL with MYC/MYCN in protein structure, Omomyc could also inhibit MYCL, representing an excellent pan-MYC family inhibitor.

To assess the potential of amplified *MYC* family genes as therapeutic target in SCLC, we investigated the effects of Omomyc on MYC inhibition in a panel of SCLC cell lines carrying genetic inactivation of *TP53* and *RB1*, as well as amplification of one of the *MYC* family genes. We show here that the inhibition of any MYC member by Omomyc induces cell growth arrest and/or apoptosis in SCLC cells even though both *TP53* and *RB1* are genetically inactivated. Notably, Omomyc also suppressed the growth of SCLC cells with *MYCL* amplification, and is able to interact with MYCL. Accordingly, we concluded that Omomyc is a pan-MYC family inhibitor, potentially useful for the treatment of SCLCs carrying any *MYC* family member amplification.

## RESULTS

### Omomyc suppresses the growth and induces death of SCLC cells

**Figure 1:**
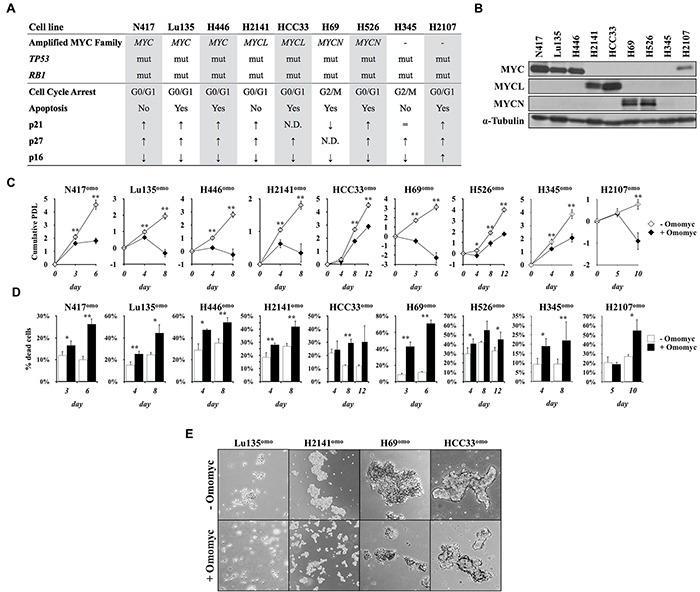
Omomyc induces growth suppression in SCLC cells **A.** Status of the MYC family genes, *TP53*, and *RB1* in SCLC cell lines used in this study. *mut: mutated.* Predominant type of the cell cycle arrest, occurrence of apoptosis and levels of p21, p27 and p16 after MYC inhibition by Omomyc are shown. **B.** Immunoblot analysis for the expression of MYC, MYCL or MYCN in SCLC cells. Media were changed 24 hr before collection of the cells. **C.** Growth curve of SCLC cells in the presence or absence of doxycycline (DX). Cumulative population doubling level (PDL) was calculated by adding the PDLs of the previous passages. Data are shown as the mean ± SD of four counts from a single representative experiment. P-values were calculated by unpaired two-tailed t-test. *p<0.05, **p<0.01. **D.** Percentage of dead cells. **E.** Representative images of floating aggregates after two (Lu135^omo^, H2141^omo^, H69^omo^) or three (HCC33^omo^) passages in culture in the presence or absence of Omomyc. Cells were photographed using phase-contrast microscopy at 5x magnitude.

To investigate the functional impact of MYC inhibition by Omomyc in SCLC cells, we established an inducible Omomyc expression system in seven cell lines carrying amplification of *MYC*, *MYCL* or *MYCN*, and two cell lines without amplification of any *MYC* family gene (Figure 1A). Both *TP53* and *RB1* are genetically inactivated in all the cell lines (Supplementary Tables 1 and 2), and the amounts of MYC proteins were higher in the cell lines carrying amplification of the respective *MYC* family gene than those without amplification of any *MYC* gene, H345 and H2107 (Figure 1B). MYC was detected in H2107, while none of the MYC proteins was detected in H345.

pTRIPZ-Omomyc-RFP contains a tetracycline response element and a CMV minimal promoter upstream the Omomyc sequence in frame with red fluorescence protein (RFP) coding sequence. Addition of doxycycline (DX) effectively induced expression of Omomyc-RFP fusion protein within 24 hr of culture (Supplementary Figure S1A), and >99% of cells showed positive RFP signals in 2-4 days (Supplementary Figure S1B). All infected cell lines expressed similar levels of Omomyc, except H69^omo^, in which its level was much higher than in other cell lines. Omomyc-RFP expression was not detected in cells cultured without DX. SCLC cell lines that conditionally expressed Omomyc were designated with the addition of “omo” to the name of each cell line.

To assess the effects of Omomyc on the growth of SCLC cell lines, the cells were serially cultured for two to three passages with and without DX (Figure 1C). Cells cultured without DX (− Omomyc) proliferated similarly to their respective parental cells. In contrast, Omomyc expression with DX addition (+ Omomyc) caused significant growth reduction in all cell lines. No toxic effects were observed by DX in non-infected parental cells (Supplementary Figure S1C). Percentages of dead cells were also increased in all cell lines after Omomyc induction (Figure 1D). Consistently, Omomyc-induced cells showed marked reduction in the size of culture aggregates (Figure 1E). Therefore, we concluded that Omomyc induced growth arrest and/or death in all tested SCLC cell lines independently of the type and extent of *MYC* family gene amplification.

### Omomyc disrupts the binding of endogenous MYC and MYCL with MAX in SCLC cells

**Figure 2:**
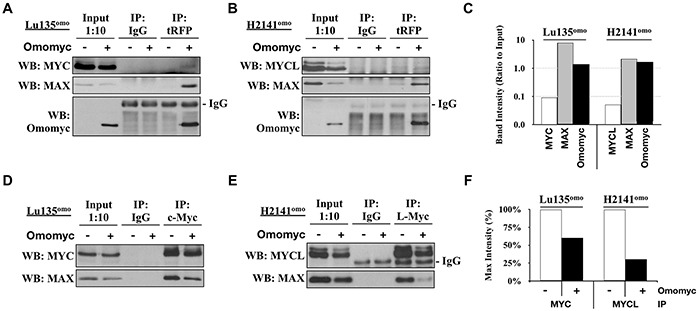
Omomyc disrupts Myc/MAX heterodimerization **A, B, D, E.** Co-immunoprecipitations (co-IP) were carried out using protein extracts from Lu135^omo^ (A, D) or H2141^omo^ (B, E) cultured in the presence or absence of Omomyc for 20 hr, using antibodies against tRFP (A, B), MYC (D), or MYCL (E). **C.** MYC, MYCL, MAX and Omomyc co-IP band intensities were quantified and normalized to corresponding Input band intensities. **F.** MAX co-IP band intensities were quantified and normalized to corresponding MYC or MYCL IP band intensities. The anti-tRFP antibody was used for Omomyc immunoprecipitation since the antibody for Omomyc crossreacted with MAX.

Omomyc has been shown to impair the DNA binding ability of the heterodimer MYC/MAX to the E-box sequence *in vitro* [18, 19], but there is no data for the effect *in vivo*. In addition, the interaction of Omomyc with MYCL as well as the effect of Omomyc on MYCL is currently unknown. Since MYC proteins are highly expressed in the SCLC cell lines used in this study, we attempted to elucidate whether Omomyc binds to both MYC and MYCL and inhibit their binding to MAX *in vivo*. Omomyc was induced in Lu135^omo^ and H2141^omo^ cells, which express high levels of MYC and MYCL accompanied by *MYC* and *MYCL* amplification, respectively. By a co-immunoprecipitation assay, it was shown that Omomyc bound to endogenous MAX and, to a less extent, to both MYC and MYCL (Figure 2A-2C), and the amount of MAX bound to MYC or MYCL was considerably reduced in the presence of Omomyc (Figure 2D-2F). Therefore, we concluded that Omomyc binds to MYC, MYCL and MAX, and efficiently hampers the heterodimerization of the endogenous MYC or MYCL with MAX in SCLC cells *in vivo*.

### Omomyc induces cell cycle arrest and apoptosis in SCLC cells

**Figure 3:**
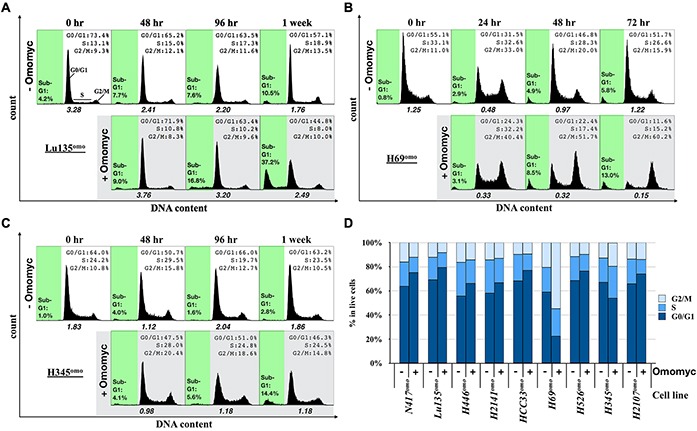
Effects of Omomyc on cell cycle progression **A**–**C**, Representative population histograms showing the cell cycle profiles and the apoptotic fractions (sub-G1) of Lu135^omo^ (A), H69^omo^ (B) and H345^omo^ (C) with or without Omomyc induction. x axis and y axis corresponds to DNA content and cell number, respectively. Sub-G1 gate is highlighted in green. The ratio of the number of cells in G1 phase versus S plus G2/M phases is shown. **D.** Cell cycle profile of cells 48 hr (N417^omo^, Lu135^omo^, H446^omo^, H69^omo^), 72 hr (H2141^omo^), 96 hr (H345^omo^), or 1 week (HCC33^omo^, H526^omo^, H2107^omo^) after Omomyc induction.

Since growth suppression was observed upon Omomyc induction in SCLC cells, we analyzed the cell cycle profile of Omomyc-induced cells at several time points after DX addition. Representative results of three cell lines, Lu135^omo^, H69^omo^ and H345^omo^, are shown in Figure 3A-3C. Differences in cell cycle distribution upon Omomyc expression became evident within 4 days of culture with DX in six out of nine cell lines (Figure 3D). In the remaining three cell lines, HCC33^omo^, H526^omo^ and H2107^omo^, differences in cell cycle distribution were observed after one week of culture (Figure 3D), likely because of their slow growth rates (Figure 1C). The percentage of cells in G0/G1 phase was increased, while those in S and G2/M phases decreased in 7 out of 9 cell lines (Figure 3D). In contrast, an accumulation of cells in G2/M phase was observed in H345^omo^ and, to a more dramatic extent, in H69^omo^ (Figure 3B-3D). Notably, the percentage of cells in sub-G1 phase also increased 1 or 2 days after cell cycle arrest in 2 *MYC*-amplifed, 1 *MYCL*-amplified, and 2 *MYCN*-amplified cell lines, suggesting the occurrence of apoptosis in these cell lines (Figure 3A-3C and Supplementary Figure S2A). By a poly ADP-ribose polymerase-1 (PARP1) immunoblot assay, increased cleavage of PARP1 was detected in the cell lines with increased sub-G1 phase, supporting the occurrence of apoptosis in these cell lines upon Omomyc induction (Supplementary Figure S2B). Apoptosis was instead not evident in N417^omo^, H2141^omo^ and H345^omo^.

### Induction of G1 arrest by Omomyc is accompanied by p21 activation

**Figure 4:**
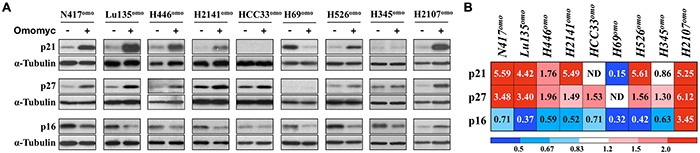
Omomyc induces CDKN1A and CDKN1B **A.** Immunoblot analysis and **B.** densitometric quantification for the expression of p21, p27 or p16 in SCLC cells with or without DX for 3-7 days. Expression levels of p21, p27 and p16 were normalized to the levels of tubulin, and the ratio of DX+ / DX- is shown. Media were changed 24 hr before collection of the cells.

Since G1 arrest was observed in the majority of cell lines after Omomyc induction, we next investigated whether it was associated with the expression of the cyclin-dependent kinase inhibitors p21 (CDKN1A), p27 (CDKN1B) and p16 (CDKN2A), which are well known modulators of G1/S transition [26]. Cells were cultured in the presence or absence of DX for 3-5 days and protein levels were evaluated (Figure 4). The amounts of p21 and p27 were increased in all cell lines that showed G1 arrest by Omomyc induction, except HCC33^omo^ (Figure 4), where p21 was not detected in either the presence or the absence of DX. In contrast, the amounts of p16 were decreased in all cell lines, except H2107^omo^. p16 reduction could be a consequence of cell cycle arrest and/or reduced E2F factors activity, since *CDKN2A* is an E2F-target gene [27]. Thus, we concluded that G1 arrest induced by Omomyc was accompanied by p21 increase in most SCLC cell lines, although *TP53*, a critical gene for *CDKN1A* transcriptional induction, is inactivated.

### The effects of Omomyc are recapitulated by MYC/MYCL silencing in SCLC cells

**Figure 5:**
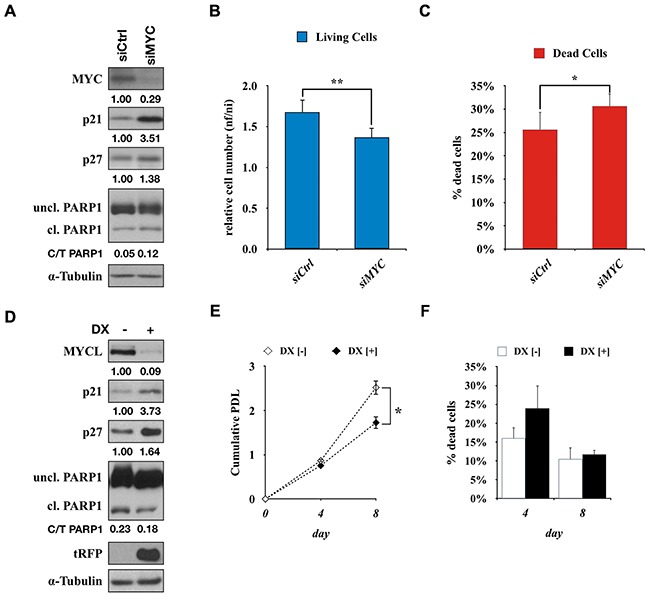
Activation of CDKN1A by knockdown of MYC or MYCL **A.** Immunoblot showing MYC, p21, p27 and PARP1 levels after 72 hr of siMYC transfection in Lu135^omo^ cells. **B**, **C.** Number of Lu135^omo^ cells and percentage of dead cells after 96 hr of transfection with control siRNA (siCtrl) or siMYC. **D.** Immunoblot showing MYCL, p21, p27, PARP1 and tRFP levels after 72 hr of doxycycline addition in H2141^shMYCL^ cells. **E**, **F.** Growth curve of H2141^shMYCL^ cells and percentage of dead cells in the presence or absence of doxycycline. *p<0.05, **p<0.01.

To test wheter the effects of Omomyc on SCLC cells were a consequence of the specific inhibition of the MYC pathway, MYC expression in Lu135^omo^ cells was suppressed using a siRNA for *MYC*. The amounts of p21 and p27, as well as the percentage of cleaved PARP1, were increased in Lu135^omo^ cells by *MYC* knockdown (Figure 5A). Cell growth reduction, as well as increase in cell death, was also observed (Figure 5B and 5C). Therefore, the effects of Omomyc and those of *MYC* inhibition by siRNA were very similar to each other, supporting that Omomyc inhibits MYC activities. Similarly, MYCL expression in H2141 cells was suppressed by using a shMYCL/RFP inducible expression vector. After 4 days of DX addition, the amount of MYCL was markedly reduced and those of p21 and p27 were increased, concomitantly with RFP induction (Figure 5D). Significant growth suppression was also observed in shMYCL treated cells (Figure 5E and 5F). Therefore, the effects of Omomyc and those of MYCL inhibition by shRNA were also very similar to each other, supporting that Omomyc efficiently inhibits MYCL activities.

### G1 arrest was not induced in H69^omo^ cells with low levels of Omomyc induction

**Figure 6:**
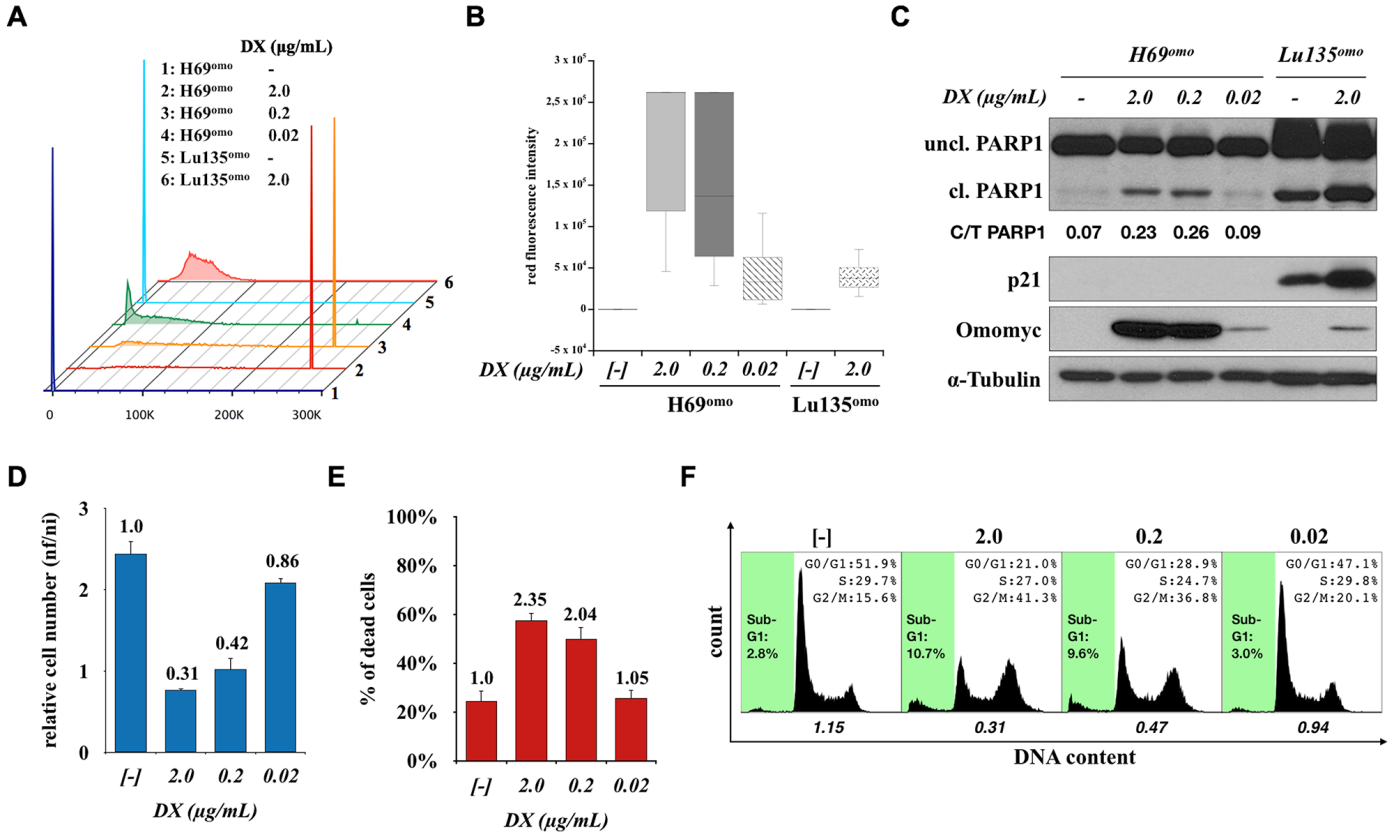
G2/M arrest in H69^omo^ induced by high levels of Omomyc expression **A**, **B.** Red fluorescence intensity in H69^omo^ induced for Omomyc-RFP expression with different concentrations of DX for 72 hr. Lu135^omo^ cells were used as a representative cell line for the induction of Omomyc-RFP in other SCLC cell lines. **C.** Immunoblot analysis of Omomyc induction, PARP1 cleavage and p21 levels. **D**, **E.** Effects of Omomyc on proliferation and death of H69^omo^. **F.** Cell cycle distribution and apoptotic fraction. The ratio of the number of cells in G1 phase versus S plus G2/M phases is shown.

Since an extremely high level of Omomyc was detected in H69^omo^ in comparison with other cell lines (Supplementary Figure S1A and S1B), we examined whether G2/M arrest induced in H69^omo^ was a consequence of a high level of Omomyc expression. We used low concentrations of DX (0.02μg/mL) to obtain Omomyc expression levels in H69^omo^ comparable to those in other cell lines (Figure 6A-6C). Effects of Omomyc on the growth and death of H69^omo^ were drastically reduced and only small differences in cell cycle distribution and apoptosis were observed. However, G1 arrest was not observed at any dose of DX addition (Figure 6D-6F). Therefore, Omomyc always induces G2/M arrest and apoptosis in H69^omo^ cells and does so in a dose dependent manner. Since G2/M arrest was also induced in H345^omo^ cells, it is likely that Omomyc effectively induces cell cycle arrest in either G1 or G2/M phase in SCLC cells (Figure 1C). These results indicate that the difference in the mode of growth suppression was not associated with Omomyc levels. The mode of cell cycle arrest was not associated with the type of amplified and overexpressed *MYC* family gene, either, since G2/M arrest was induced in one of the two *MYCN* amplified cell lines, H69^omo^, and in one of the two cell lines without amplification of any *MYC* family gene, H345^omo^. However, the effect of Omomyc seemed to be reduced in a *MYC* non-amplified cell line, H345^omo^, where none of the MYC proteins were detected by Western Blot analysis (Figure 1B). In H345^omo^, neither apoptosis nor PARP1 cleavage was observed. In contrast, the effect of Omomyc in another *MYC* non-amplified cell line, H2107^omo^, was similar to *MYC* amplified cell lines. In H2107^omo^, a considerable level of MYC was expressed despite the lack of amplification of any *MYC* family gene (Figure 1B). Therefore, it is possible that SCLC cells with *MYC* family gene amplification and/or overexpression are more addicted to MYC for their growth and survival than the cells without *MYC* amplification and/or overexpression.

### Induction of G1 arrest by Omomyc is dependent on P21 activation

**Figure 7:**
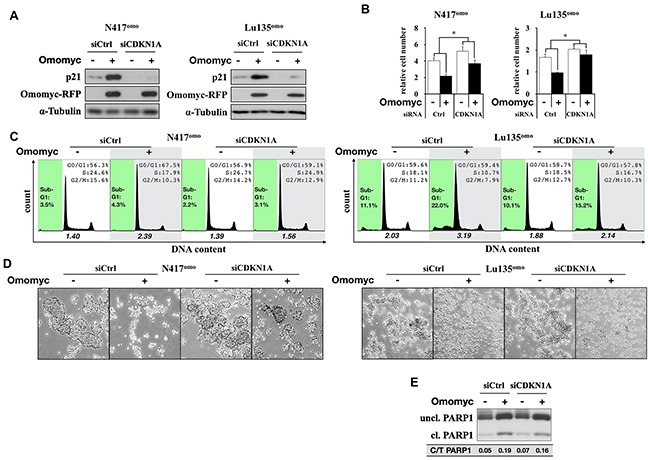
Downregulation of p21 impairs the Omomyc-induced G1 arrest **A.** Immunoblot analysis for the knock-down of p21 in N417^omo^ and Lu135^omo^ cells. Cells were cultured with or without DX (+/− Omomyc). **B.** Number of cells after 72 hr (N417^omo^) or 96 hr (Lu135^omo^) of culture. Data are shown as mean ± SD (n=4). P-values were calculated by unpaired two-tailed t-test. *p<0.01. **C.** Cell cycle distribution after after 72 hr (N417^omo^) or 96 hr (Lu135^omo^) of culture. x axis and y axis corresponds to DNA content and cell number, respectively. Sub-G1 gate is highlighted in green. The ratio of the number of cells in G1 phase versus S plus G2/M phases is shown. **D.** Representative images of floating cell aggregates after 72 hr (N417^omo^) or 96 hr (Lu135^omo^) of culture. Cells were photographed using phase-contrast microscopy at 5x magnitude. **E.** Cleavage of PARP1 in Lu135^omo^ was evaluated by immunoblot analysis. Band intensity was quantified by densitometry and the ratio of cleaved/total (uncleaved+cleaved) was calculated.

We previously reported that exogenous p21 expression induced G1 arrest in N417 cells [10], while it was shown that the growth of SCLC cells was not suppressed by over-expression of p27 [28]. Therefore, we next investigated whether G1 arrest and cell death induced by Omomyc were dependent upon p21 activation, encoded by the *CDKN1A* gene. N417^omo^ cells, which underwent G1 arrest by Omomyc, and Lu135^omo^, which showed both G1 arrest and apoptosis by Omomyc, were treated with a siRNA for *CDKN1A* at the time of Omomyc induction. After 72 hr of induction, the amount of p21 increased in both control cell lines (siCtrl), whereas such an increase was suppressed in the respective *CDKN1A* knocked-down (CDKN1A^kd^) cells (siCDKN1A in Figure 7A). The effect of Omomyc on growth suppression was significantly reduced in both cell lines by *CDKN1A* knockdown (Omomyc [+] vs [−]; N417^omo^ siCtrl 54.6%, siCDKN1A 71.1% - p<0.01, n=4; Lu135^omo^ siCtrl 58.6%, siCDKN1A 87.6% - p<0.01, n=4) (Figure 7B). Consistently, G1 arrest and the reduction in the size of cell aggregates were less evident after Omomyc induction in CDKN1A^kd^ cells in comparison with siCtrl cells (Figure 7C and 7D). Therefore, the lack of *CDKN1A* induction hampered the effects of Omomyc on both cell growth and cell cycle progression. These results indicate that Omomyc induces G1 arrest through the activation of p21. However, the increase in sub-G1 fractions, as well as that in cleaved PARP1, caused by Omomyc was not affected in Lu135^omo^ CDKN1A^kd^ cells (Figure 7C and 7E).

### Activation of CDKN1A requires high levels of TP73

**Figure 8:**
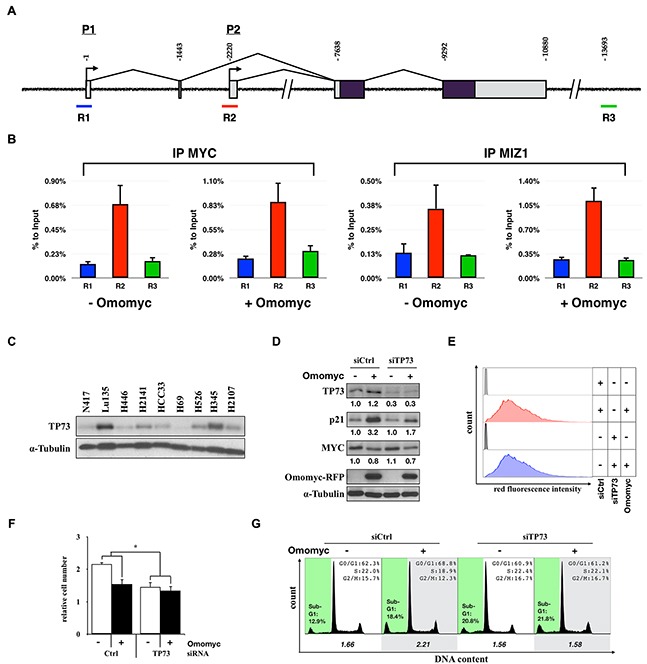
Activation of CDKN1A by TP73 in a MYC-amplified SCLC cell line **A.** Schematic representation of the *CDKN1A* gene and sequences amplified after chromatin immunoprecipitation (ChIP). Binding of MYC and MIZ1 has been reported in the proximity of P2 transcription starting site, spanning R2 region. **B.** ChIPs were carried out using chromatin obtained from Lu135^omo^ cultured in the presence or absence of Omomyc for 48 hr using antibodies against MYC or MIZ1. R1 and R3 regions were used as negative controls of DNA: protein binding. **C.** Immunoblot analysis for the expression of TP73 in SCLC cells. **D.** Immunoblot analysis for the expression of TP73, p21, MYC, and Omomyc in TP73 knock-down Lu135^omo^ cells 72 hr after Omomyc induction. Protein levels were normalized to the levels of tubulin. **E.** Flow cytofluorimetric analysis of Omomyc-RFP red fluorescence (575nm ±13) after 96 hr of DX addition. **F.** Number of cells after 96 hr from the induction of Omomyc. Data are shown as the mean ± SD (n=3). P-values were calculated by unpaired two-tailed t-test. *p<0.05. **G.** Cell cycle distribution. The ratio of the number of cells in G1 phase versus S plus G2/M phases is shown.

Since increased levels of p21 were required for Omomyc-induced G1 arrest, we next investigated the molecular mechanism of *CDKN1A* activation in the absence of functional *TP53*. Since the binding of MYC to the promoter of *CDKN1A* through heterodimerization with MIZ1 is a well-established mechanism of *CDKN1A* repression by MYC [16, 29], we further investigated the effect of Omomyc on the binding of MYC and MIZ1 to the *CDKN1A* promoter by chromatin immunoprecipitation assay. The binding of MYC and MIZ1 to the *CDKN1A* promoter was confirmed; however, the binding of both proteins was not inhibited by Omomyc (Figure 8A and 8B).

TP53 belongs to a family of proteins, including TP63 and TP73, that increase the expression of similar groups of genes through the direct binding within their promoter regions (such as *CDKN1A*) which, in turns, induce cell cycle arrest, senescence, and/or apoptosis [30]. It was reported that MYC can repress TP73-mediated transcriptional activation of *CDKN1A*, and that Prefoldin subunit 5 (*PFDN5*), also known as MYC binding protein 1 (*MM1*), antagonizes the inhibitory effect of MYC on TP73-dependent transactivation [31]. In SCLCs, expression of *TP73* is relatively high [32], whereas expression of *TP63* and *MM1* is low (Supplementary Figure S3). Therefore, we evaluated TP73 protein levels in several representative cell lines. The amounts of TP73 were different among them (Figure 8C). Interestingly, TP73 was highly expressed in Lu135^omo^, where p21 was also highly induced by Omomyc. Therefore, we investigated whether p21 activation by Omomyc was dependent on TP73 in this cell line. Lu135^omo^ cells were treated with a siRNA for *TP73* and Omomyc was induced. The level of p21 up-regulation by Omomyc was reduced in *TP73* knockdown (TP73^kd^) cells (Figure 8D). Similar levels of Omomyc-RFP were detected in control and TP73^kd^ Omomyc-induced cells, indicating that different p21 levels were not due to different Omomyc expression levels (Figure 8D and 8E). Then, the effects of Omomyc on cell growth and cell cycle profile were evaluated. The effect of Omomyc on growth reduction was decreased in TP73^kd^ cells (Figure 8F) (Omomyc [+] vs [−], siCtrl 71.1%, siTP73 90.9%, p<0.05, n=3). No differences on cell cycle distribution were detected either (Figure 8G). These results indicate that high levels of TP73 are required for both p21 activation and G1 arrest induction by Omomyc in Lu135^omo^ cells. Therefore, it is possible that Omomyc relieves the repressive role of MYC on the transcriptional activation of *CDKN1A*, which is activated in part by TP73 in SCLC cells.

## DISCUSSION

**Figure 9:**
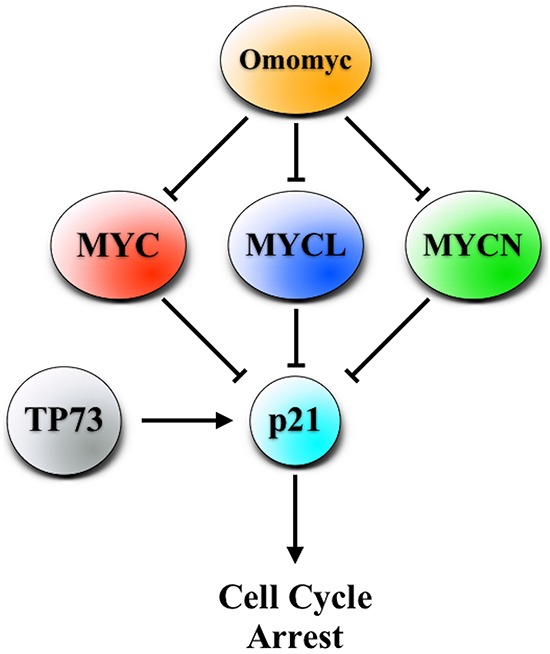
MYC, MYCL and MYCN inhibition by Omomyc induces cell cycle arrest through the activation of p21, in some cases through the TP73 pathway

Cell cycle arrest, differentiation, senescence or cell death have been reported to occur in cancer cells after MYC inhibition, through different molecular mechanisms [25, 33–35]. It is likely that MYC inhibition affects different molecular pathways in cancer cells based on cell types, accumulated genetic alterations, and degree and mode of the inhibition. Here, we show that MYC inhibition by Omomyc, a dominant-negative MYC, suppresses the growth of SCLC cells with *TP53* and *RB1* inactivation carrying *MYC*, *MYCL*, or *MYCN* amplification. Occurrence of cell cycle arrest in G1 phase was the consequences of MYC inhibition in most SCLC cell lines, indicating that *MYC* family genes amplified SCLC cells are addicted to MYC proteins function for their growth (Figure 9). Therefore, MYC family gene products appear promising targets for the treatment of SCLC patients. Since one of the three *MYC* family genes is amplified in a mutually exclusive manner in 20% of SCLCs, the development of therapeutic strategies against any MYC family members will be highly useful in the treatment of a significant fraction of SCLC.

Omomyc has been shown to suppress the growth of several cancer cells through its activity as a MYC inhibitor [22, 23, 25]. In this study, Omomyc also suppressed the growth of SCLC cells with high expression of MYC, MYCN or MYCL. Previously, Omomyc was shown to inhibit the binding of MYC and MYCN to MAX [18, 20]. Here we show that Omomyc also inhibits the binding of MYCL to MAX in SCLC. These data strengthen the evidence indicating that Omomyc represents a common inhibitor for the interaction of MAX with all MYC proteins *in vivo* and further supports the utility of Omomyc as a therapeutic strategy for SCLC. In each SCLC cell line used in this study, only one of MYC proteins was highly expressed, but two or three MYC proteins are often co-expressed in SCLC cells [14]. Therefore, Omomyc will be also highly effective in SCLC cells in which more than two MYC proteins are simultaneously expressed.

Omomyc consistently and remarkably increased the levels of p21, encoded by the *CDKN1A* gene, in most SCLC cell lines. Therefore, it is likely that Omomyc relieves the expression of genes whose transcription is repressed by MYC proteins. The present results further indicate that MYCL, similar to MYC and MYCN, also represses the transcription of the *CDKN1A* gene. However, Omomyc did not suppress the binding of MYC and MIZ1 to the promoter region of *CDKN1A* (Figure 8A and 8B), consistently with the previous observation [20]. It is possible that Omomyc substitutes MAX as a scaffold for the MYC-MIZ1 interaction. It is also possible that MAX is not strictly necessary for the formation of a MYC-MIZ1 functional complex to the *CDKN1A* promoter. The lack of Omomyc acrivity to inhibit the binding of MYC-MIZ1 complex to the *CDKN1A* promoter indicates that Omomyc interferes with another MYC-dependent mechanism of *CDKN1A* regulation, such as the activation of MYCLos non-coding RNA [36].

Inactivation of *TP53* could interfere with therapeutic approaches based on MYC targeting, because of the opposite roles of MYC and TP53 in the regulation of p21. MYC hampers p21 functions in several contexts, for instance by repressing its transcription [37, 38], whereas TP53 is a transcriptional activator of *CDKN1A* [29, 39]. Therefore, MYC inhibition would allow the TP53-dependent activation of *CDKN1A*, which in turns leads to cell cycle arrest and apoptosis. However, the *TP53* gene is mutated in all the SCLC cell lines used in this study, indicating that p21 up-regulation occurs in a TP53-independent manner. Therefore, we pursued the possible involvement of TP73, another TP53 family protein, in the up-regulation of p21 in SCLC cells after Omomyc induction. At least in the Lu135 cell line, TP73 was required for the activation of p21 and for induction of G1 arrest (Figure 8 and Figure 9). It was reported that MYC abrogates TP73-mediated activation of TP53-target genes by direct protein:protein interaction [31, 40]. Therefore, it is possible that MYC also abrogates the TP73 mediated transcriptional activation of the *CDKN1A* gene in a subset of SCLCs. However, cell growth was also reduced after knockdown of TP73 in cells without Omomyc, indicating that high levels of TP73 are required for cell survival (Figure 8D). Previously, it was reported that TP73 alpha has an anti-apoptotic effect in SCLC cells, whereas TP73 beta has a pro-apoptotic effect [41]. Since both isoforms are targeted by the treatment with siRNA, it was likely that apoptosis was induced by the reduced TP73 alpha expression independently of Omomyc (Figure 8E). In this study, we found that different levels of TP73 are expressed among the SCLC cell lines. A recent study revealed that *TP73* is mutated, deleted and rearranged in ~13% of SCLC cases [42]. Therefore, the effects of MYC inhibition could vary according to the status of the *TP73* gene and its expression. Further studies are needed to clarify the interconnection between MYC proteins and TP73 in SCLC cells.

Occurrence of G1 arrest and apoptosis by MYC inhibition in *TP53* inactivated cells have been reported in *RB1* wild-type cells with high levels of MYC or MYCN expression [35, 43, 44]. Moreover, in *TP53* inactivated melanoma cells, inhibition of *RB1* was reported to prevent the occurrence of apoptosis induced by MYC inhibition [45]. In this study, both growth arrest and apoptosis were induced by MYC inhibition in SCLC cells with *RB1* mutations. CDKIs, such as p21 and p27, are well known modulators of RB1 phosphorylation through the inhibition of the activity of CDK-cyclin complexes: CDKIs induce cell cycle arrest by maintaining RB family proteins, RB1, p107 (RBL1) and Rb2/p130 (RBL2), in a hypophosphorylated state, which in turns repress the transcription of E2F-target genes and block transition from G1 to S phase [26, 27]. Since *RB1* is genetically inactivated and *RBL2* has been reported to be transcriptionally repressed in SCLC cells, it is possible that G1 arrest was mediated by the hypophosphorylation of p107 [46–48]. Alternatively, it is also possible that p21 directly interacts with E2F1 and PCNA, and these interactions induce G1 arrest in SCLC cells without functional RB family proteins [37]. A recent study also revealed that *RBL1* and *RBL2* are mutated in a subset of SCLCs together with *TP53* and *RB1* and mutually exclusively with each other and with *TP73* [42]. Indeed, in two of the nine SCLC cell lines examined in this study, H69 and H345, G2/M arrest, but not G1 arrest, was induced by Omomyc induction. Therefore, it is likely that the effect of Omomyc could be different among SCLCs due to accumulated genetic alterations of cell cycle regulators other than *TP53* and *RB1*.

In summary, we demonstrated here that MYC inhibition by Omomyc induces cell cycle arrest in all the nine SCLC cell lines tested. The results strongly indicate that SCLC cells are addicted to MYC proteins for their growth and, therefore, are highly sensitive to MYC inhibition for their growth suppression in spite of the presence of *TP53* and *RB1* genetic inactivation. Accordingly, it was concluded from this study that MYC inhibition would be a promising therapeutic strategy for a significant fraction of SCLC, an aggressive cancer with extremely high mortality rate.

## MATERIALS AND METHODS

### Cell cultures

SCLC cell lines, N417, H2141, HCC33, H69, H345 and H2107, were obtained from Dr. J. D. Minna (University of Texas Southwestern, Dallas), H526 from Dr. C. C. Harris (NCI, Bethesda), Lu135 from Dr. T. Terasaki (National Cancer Center, Tokyo, Japan), and H446 from the Japanese Collection of Research Bioresources. Cells were cultured in RPMI supplemented with L-Glutamine and HEPES supplemented with 10% tetracycline-free FBS (Clontech) with 5% CO_2_ at 37°C.

### Lentivirus preparation, infection and selection of infected cells

Construction of the lentiviral vector pTRIPZ-Omomyc-RFP for the inducible expression of a Omomyc-RFP fusion protein was described previously [25]. A pTRIPZ-shMYCL-RFP expression vector, V2THS197161, was purchased from GE Healthcare Dharmacon. For lentivirus production, 293T cells were plated and medium containing 25μM chloroquine was added on the following day. Two hours later, cells were transfected with pTRIPZ-Omomyc-RFP or pTRIPZ-shMYCL-RFP, plus psPAX2 and pMD.G2, using the calcium phosphate method. After 16 hr, the medium was replaced with RPMI supplemented with tetracycline-free FBS. After 24 hr and 48 hr, first and second aliquots of media were collected and filtered with a 0.45μm PVDF filter. Cells were infected with the virus for 24 hr with the addition of polybrene (2μg/ml). Puromycin was added to kill non-infected cells and to maintain optimal plasmid integration.

### Cell growth assays

Cell growth assays were performed with a modification of serial cultivation method [49]. Briefly, cells at the concentration of 5.0×10^4^/ml (H446), 1.0×10^5^/ml (N417, Lu135, H2141, HCC33, H526, H69, H345), or 2.0×10^5^/ml (H2107) were plated and replated at the same density every 3 days (N417, H69), 4 days (Lu135, H446, H2141, HCC33, H526, H345), or 5 days (H2107) for two passages. Number of days between each passage was chosen to allow at least one population doubling in untreated cells. Cell number and viability were determined by dye exclusion trypan blue assay using Cell Countess® (Life Technologies). Population doubling level (PDL) was calculated using the formula PDL = log_2_ (Nf/Ni), where Ni is the initial number and Nf is the final number of cells.

### Transfections

siRNA was transfected using RNAiMAX (Life Technologies). Concentrations of siRNA and RNAiMAX were 50nM and 2.5μl/ml, respectively. siRNA sequences (ON-TARGETplus siRNA, GE Dharmacon) used were: MYC (pool of 2 sequences: J-003282-25, J-003282-26), CDKN1A (J-003471-12), and TP73 (J-003331-10). After 6 hr, media was changed and, if required, doxycycline was added. A non-targeting siRNA was used as control (D-001600-01, GE Dharmacon). Control sequence, containing DY-547 red fluorescent oligonucleotides, was used to estimate transfection efficiency. Cells were analyzed after 72-96 hr of transfection.

### Cell cycle analysis

Cells were centrifuged at 200g for 5 min, washed in PBS, counted, and fixed with 70% ice-cold ethanol. Fixed cells were centrifuged at 1000g for 5 min, washed, resuspended in PBS containing 0.1% Triton X-100, 0.2mg/ml RNase A and 1μg/ml DAPI, and analyzed with FACS Fortessa (Beckton Dickinson). At least 20,000 events were tested to evaluate cell cycle status. The percentage of cells in sub-G1 (<2n) was calculated based on the DNA content, and those in G1, S, and G2/M were calculated in living cell population based on their DNA content/FSC-A profile.

### Western blot analysis

Cells were lysed in buffer (50mM TRIS, 0.5% sodium deoxycholate, 1.0% NP-40, 0.1% SDS, 150mM NaCl, 2mM EDTA) supplemented with protease inhibitors (Roche). Lysates (15-30μg) were resolved by SDS-PAGE, transferred to nitrocellulose membranes, and probed with the following antibodies: MYC (sc-40, Santa Cruz), MYCL (#AF4050, R&D), MYCN (#9405, Cell Signaling), Omomyc, p21 (#2947, Cell Signaling), p27 (sc-1641, Santa Cruz), p16 (51-1325GR, BD), PARP1 (#9542, Cell Signaling), TP73 (sc-7957, Santa Cruz), α-Tubulin (CP06, CalBiochemicals). Membranes were then incubated with a peroxidase-conjugated antibody. Enhanced chemiluminescence was performed according to manufacturer's instructions (Western Lightning Plus, Perkin Elmer).

### Co-immunoprecipitation assay

Co-immunoprecipitation assays were performed according to manufacturer's instructions (Dynabeads Protein A, Life Technologies). Whole cell extracts were obtained by incubating cells in 1.0% NP-40, 250mM NaCl, 50mM Tris-HCl (pH 7.4), 5mM EDTA, 0.02% NaN_3_ buffer supplemented with protease and phosphatase inhibitors (Roche). Extracts were pre-cleared with 20μl of beads for 1 hr, and 0.5mg of proteins were incubated for 1 hr with 50μl of beads pre-incubated with 1μg/ml of the following antibodies: anti-tRFP (AB234, Evrogen), anti-MYC (N-262, Santa Cruz), anti-MYCL (AF4050, R&D Systems), anti-MAX (C-17, Santa Cruz Biotechnology). Samples were washed with PBS containing 0.1% Tween-20, eluted with Nu-Page LDS and Reducing Sample Buffers (Life Technologies), and loaded on a 10% acrylamide gel.

### Cromatin immunoprecipitation assay

ChIP assays were carried out according to manufacturer's instructions (ChIP-IT Express Enzymatic Kit, Active Motif). The following antibodies were used: anti-MYC (N-262, Santa Cruz) or anti-MIZ1 (N-17, Santa Cruz). For the amplification of R1, R2 and R3 regions, the following previously reported primers were used: 5′-AGCAGGCTGTGGCTCTGATT-3′ (R1, Forward), 5′-CAAAATAGCCACCAGCCTCTTCT-3′ (R1, Reverse), 5′-ACCGGCTGGCCTGCTGGAACT-3′ (R2, Forward), 5′-TCTGCCGCCGCTCTCTCACCT-3′ (R2, Reverse), 5′-TCTGTCTCGGCAGCTGACAT-3′ (R3, Forward), 5′- ACCACAAAAGATCAAGGTGAGTGA-3′ (R3, Reverse). Each sample was used as template in Real-Time PCR to evaluate the enrichment of R1, R2 or R3 regions.

## SUPPLEMENTARY MATERIAL FIGURES AND TABLES


