# Supplementary material for: Growth suppression by MYC inhibition in small cell lung cancer cells with TP53 and RB1 inactivation
Source: Oncotarget. 2016 Apr 18;7(21):31014–28. doi: 10.18632/oncotarget.8826 (PMC5058735; doi:10.18632/oncotarget.8826)
Supplement: Supplementary file 1 [file oncotarget-07-31014-s001.pdf]

SUPPLEMENTARY FIGURES AND TABLES

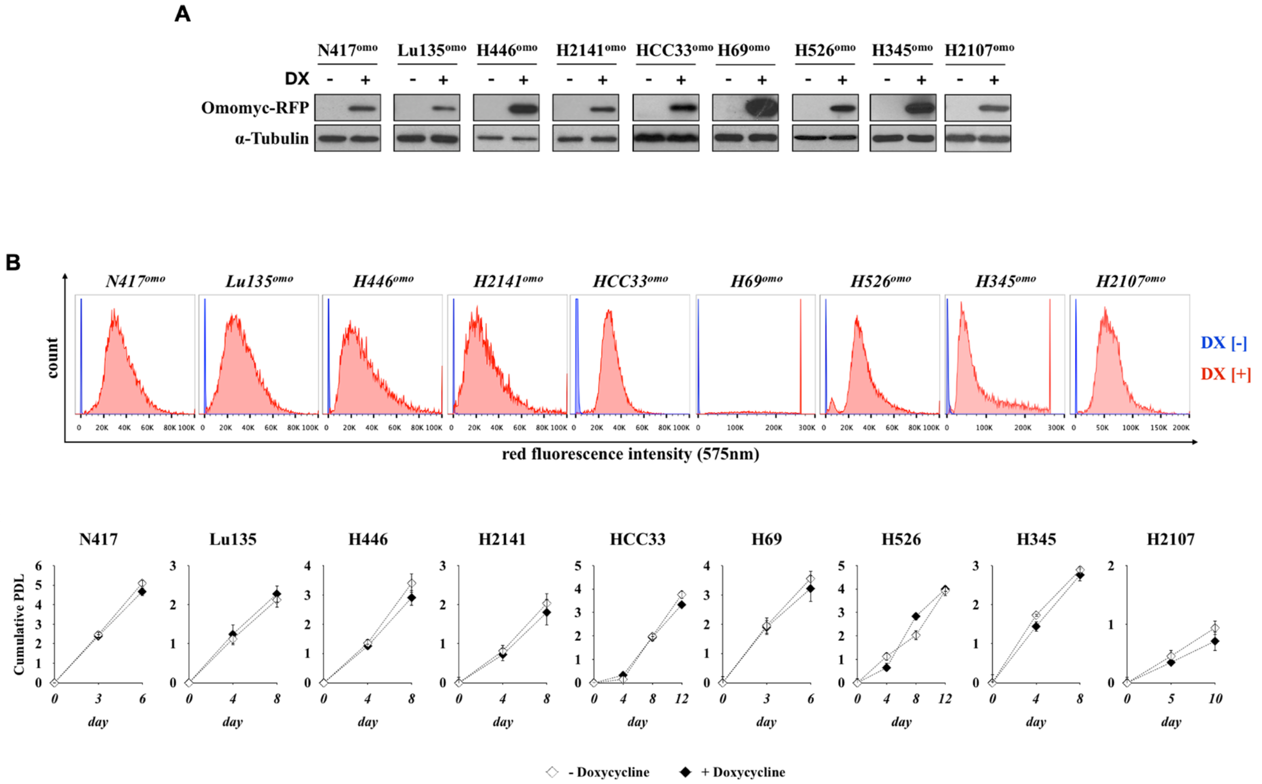

Supplementary Figure S1: Induction of Omomyc in SCLC cell lines. A. Immunoblot analysis of Omomyc-RFP in cells after 24 hr of doxycycline (DX) addition to culture medium. **B.** Flow cytofluorimetric analysis of red fluorescence (575nm ±13) after 48 hr (N417, Lu135, H446, H2141, H69, H345), 72 hr (H2107) or 96 hr (HCC33, H526) of DX addition. **C.** Effects of DX on the growth of parental SCLC cell lines. The graphs show the population doubling level (PDL) of each cell line with or without DX.

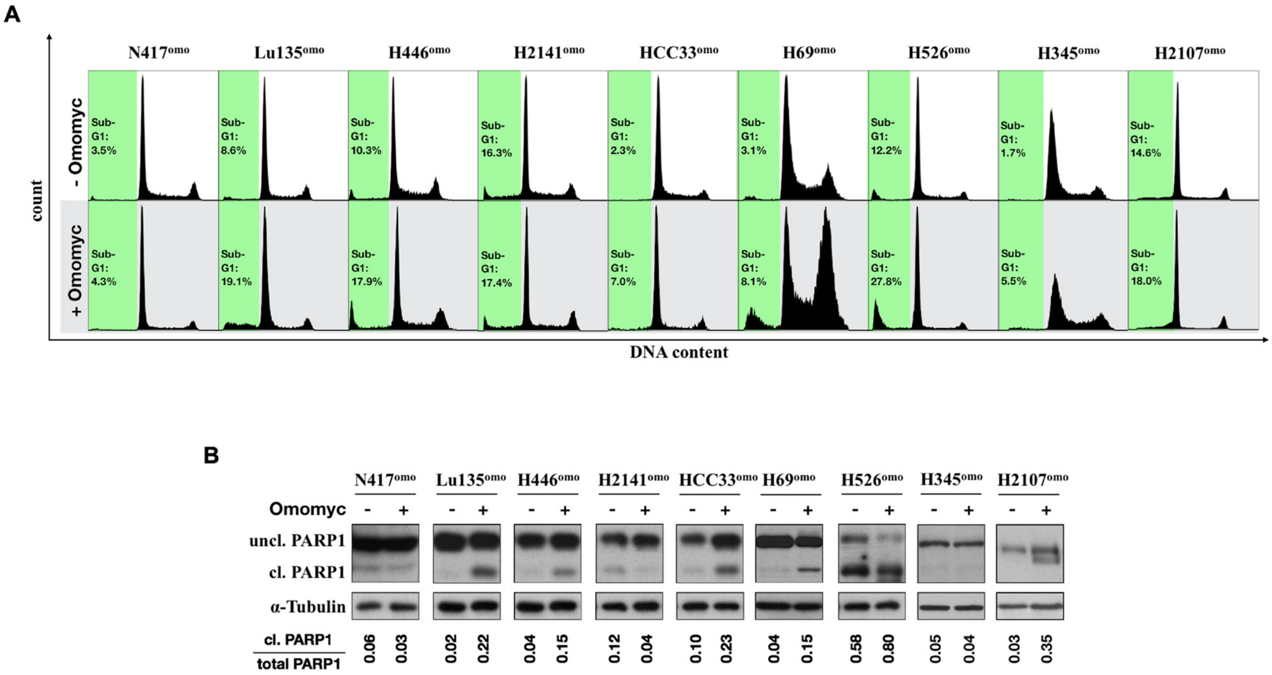

Supplementary Figure S2: Omomyc induces apoptosis. A. Percentage of cells in sub-G1 phase after 3 days (N417omo, H69omo), 96 hr (Lu135omo, H446omo, H2141omo, H345omo), or 1 week (HCC33omo, H526omo, H2107omo) of Omomyc induction. **B.** Immunoblot analysis of PARP1 after one passage in culture in the presence or absence of Omomyc. Band intensity was quantified by densitometry and the ratio of cleaved/total (uncleaved+cleaved) PARP1 was calculated.

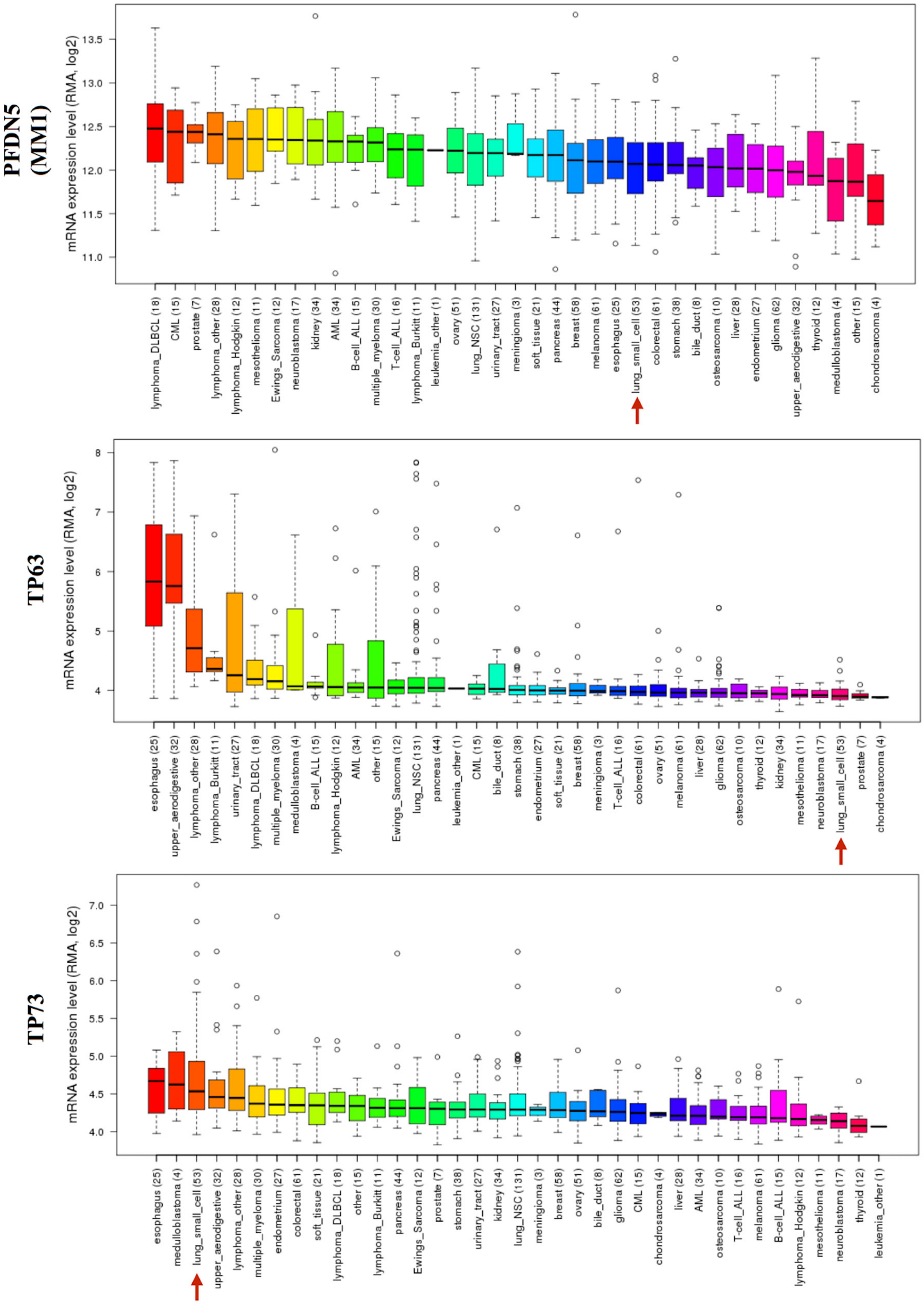

Supplementary Figure S3: Meta-analysis of TP73, TP63, and MM1 mRNA expression. Query of mRNA expression from the Cancer Cell Line Encyclopedia (CCLE). The numbers in parenthesis along the y-axis indicate the number of tumor cell lines used for each sample set. RMA, robust multi-array average; NSC, non-small-cell; CML, chronic myelogenous leukemia; ALL, acute lymphoblastic leukemia; DLBCL, diffuse large B-cell lymphoma; AML, acute myelogenous leukemia. Small cell lung cancer is indicated by red arrows.

Supplementary Table S1: TP53 status in SCLC cell lines used in this study

Cell line	Type of mutation	DNA sequence change	Amino acid change	Pubmed Id
N417	Nonsense	c.892G>T	p.E298*	1565469; 22941189
Lu135	Missense	c.730G>T	p.G244C	1312700
H446	Missense	c.461G>T	p.G244C	15188009
H2141	Nonsense	c.625A>T	p.R209*	1312696
HCC33	Missense	c.725G>A	p.C242Y	22460905
H69	Nonsense	c.511G>T	p.E171*	1648702
H526	Splice site	c.97-1G>C	-	2164047; 15188009
H345	Missense	c.707A>G	p.Y236C	15188009
H2107	Nonsense	c.491T>A	p.K101*	22941189

Supplementary Table S2: RB1 status in SCLC cell lines used in this study

Cell line	Type of mutation	DNA sequence change	Amino acid change	Pubmed Id
N417	Splice site	c.2686+1G>A	-	22941189; 2159370
Lu135	Frameshift	c.1980_1983delCCGG	p.R661fs*1	15188009
H446	Splice site	c.2127-2A>T	-	15188009
H2141	Splice site	c.1499-2A>G	-	15188009
HCC33	Frameshift	c.1202delT	p.S402fs*8	15188009
H69	Nonsense	c.2242G>T	p.E748*	15188009; 2159370
H526	Nonsense	c.91G>T	p.E31*	15188009; 2159370
H345	Deletion - Intronic Site	c.106850_118816del	-	15188009
H2107	Splice site; Frameshift	c.1381+1G>T; c.583_584insT	p.W195fs*8	15188009; 22941189
